# Quilopericárdio Primário Idiopático

**DOI:** 10.36660/abc.20260066

**Published:** 2026-07-31

**Authors:** Camila Helena Ruffo, Alfredo Aurélio Marinho Rosa, Guilherme Lima Favaro, Juliana Sayuri Maia Hirose, Raissa Matias Lewinter, Natan Melillo, Leonardo Izzo Silva, Ramez Anbar, Murilo Martins Coelho, Giuliano Generoso

**Affiliations:** 1 Hospital Sírio - Libanês São Paulo SP Brasil Hospital Sírio - Libanês, São Paulo, SP – Brasil

**Keywords:** Pericárdio, Derrame Pericárdico, Diagnóstico por Imagem

## Introdução

O quilopericárdio é uma entidade rara, definida pelo acúmulo de quilo no espaço pericárdico caracterizando-se por elevada concentração de triglicerídeos e predomínio linfocitário. A primeira descrição foi em 1954 e desde então essa condição permanece pouco frequente, com a maior parte das evidências provenientes de relatos de caso e pequenas séries, o que dificulta o reconhecimento precoce e a padronização do manejo.

As principais causas incluem linfangiectasia congênita, lesão iatrogênica após cirurgia cardíaca ou torácica, neoplasias, traumas, infecção, radiação, hipertensão pulmonar, trombose de veia subclávia e causas primárias ou idiopáticas. O quilopericárdio primário, tem incidência variável na literatura e constitui diagnóstico de exclusão.

Relatamos um caso de quilopericárdio primário em paciente jovem, enfatizando os desafios diagnósticos e terapêuticos dessa condição rara.

## Descrição

Paciente do sexo feminino, 31 anos, previamente hígida, natural e procedente de São Paulo (SP), procurou atendimento médico, em contexto ambulatorial, com queixa de cansaço progressivo, fadiga e intolerância ao esforço, iniciado há 5 meses. Relatava dispneia aos esforços mínimos, associada a tontura, sensação de desequilíbrio e sudorese diurna, com piora gradual ao longo dos meses. Negava dor torácica, febre, tosse, emagrecimento, sintomas infecciosos ou história de trauma torácico ou cirurgias prévias. De antecedentes pessoais, apresentava bronquite e estava em investigação de amenorreia primária, com suspeita de síndrome de Mayer–Rokitansky–Küster–Hauser. Negava tabagismo, etilismo ou uso de drogas ilícitas.

No exame físico inicial paciente mostrava-se em bom estado geral, com PA:129x67 mmHg, FC:65 bpm, FR:16 mrpm, saturação de oxigênio 97% em ar ambiente, afebril, sem sinais de congestão sistêmica, com exame cardiovascular e respiratório sem alterações significativas.

A investigação inicial incluiu eletrocardiograma, exames laboratoriais, sem achados específicos, e uma radiografia de tórax (Figura 1) com evidente aumento da silhueta cardíaca. A partir desse achado, foi solicitado ecocardiograma transtorácico, que evidenciou derrame pericárdico de moderado volume, sem sinais de tamponamento cardíaco.

**Figura 1:**
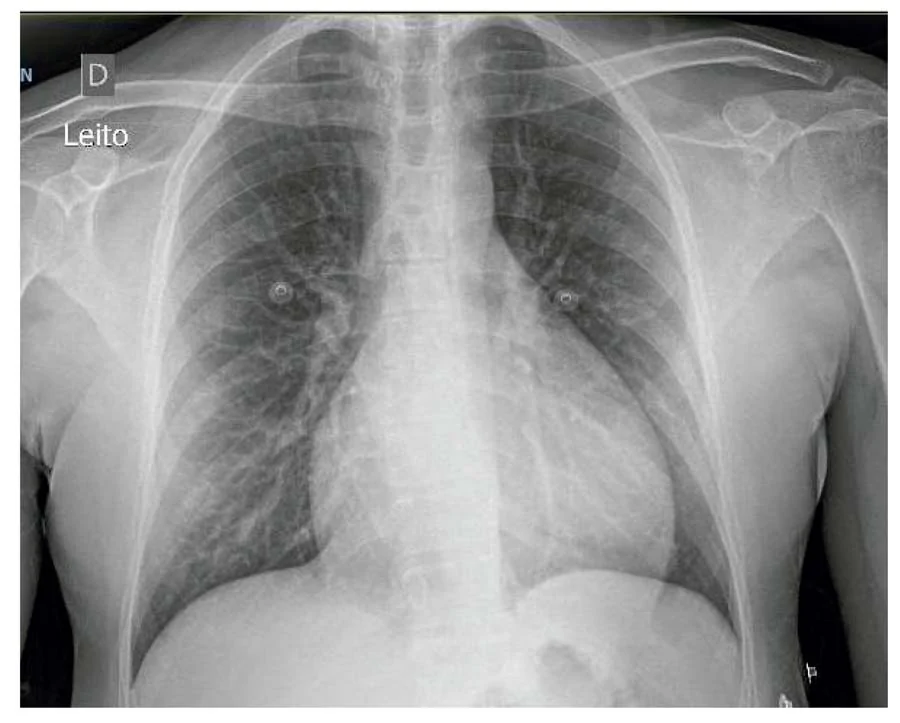
– RX de tórax: aumento da silhueta cardíaca.

Optado assim por internação hospitalar para investigação de etiologia do derrame pericárdico com exames laboratoriais como provas reumatológicas, infecciosas, metabólicas e exames de imagem complementar.

A análise complementar mostrou hemograma, eletrólitos, parcial de urina, função renal, hepática e tireoidiana normais, além de marcadores inflamatórios discretamente alterados: PCR:1,02 mg/dL(VR<0,5mg/dL) e VHS:41 mm (VR:2-20mm). A avaliação reumatológica demonstrou alterações inespecíficas e discretas, as quais, não determinam diagnóstico reumatológico (Tabela 1).

**Tabela 1:** – Resultados dos exames de investigação reumatológica

Exames	Resultados	Valor de referência
FAN	negativo	negativo
FR	negativo	negativo
C3	145mg/dL	81-157mg/dL
C4	46mg/dL	13-39mg/dL
ASLO	592UI/mL	<200UI/mL
Anticoagulante lúpico	negativo	negativo
Anti-DsDNA	negativo	negativo
Anti-Jo 1	negativo	negativo
Anti-SCL 70	negativo	negativo
Anti-SSBLA	negativo	negativo
Anti-SSBRo	negativo	negativo
Anti-Sm	negativo	negativo
Anti-RNP	negativo	negativo
Anticardiolipina	negativo	negativo
Alfa-1 glicoproteína ácida	73mg/dL	50-120mg/dL
ANCA	negativo	negativo
Creatinina urinária	20mg/kg/dia	11-20mg/kg/dia
Eletroforese de proteína		
Albumina	53%	55-66%
Beta-globulina	6,5%	3,2-6,5%
Gama-globulina	21%	11-18%

FAN: fator antinúcleo; FR: fator reumatoide; C3: complemento C3, C4: complemento C4; ASLO: antiestreptolisina O; Anti-DSDNA: anticorpo anti DNA nativo; Anti-Jo1: anticorpo anti- Jo1; Anti-SCL70: anticorpo anti-SCL70; Anti-SSBLA: anticorpo anti SSBLA: Anti-SSARo: anticorpo anti SSARo; Anti-SM: anticorpo anti SM; Anti-RNP: anticorpo anti RNP; ANCA: anticorpos anti- citoplasma de neutrófilos.

Para melhor definição anatômica, foi submetida a ressonância magnética cardíaca que demonstrou função sistólica biventricular preservada, ausência de fibrose miocárdica e presença do derrame pericárdico sem outras alterações (Figura 2).

**Figura 2:**
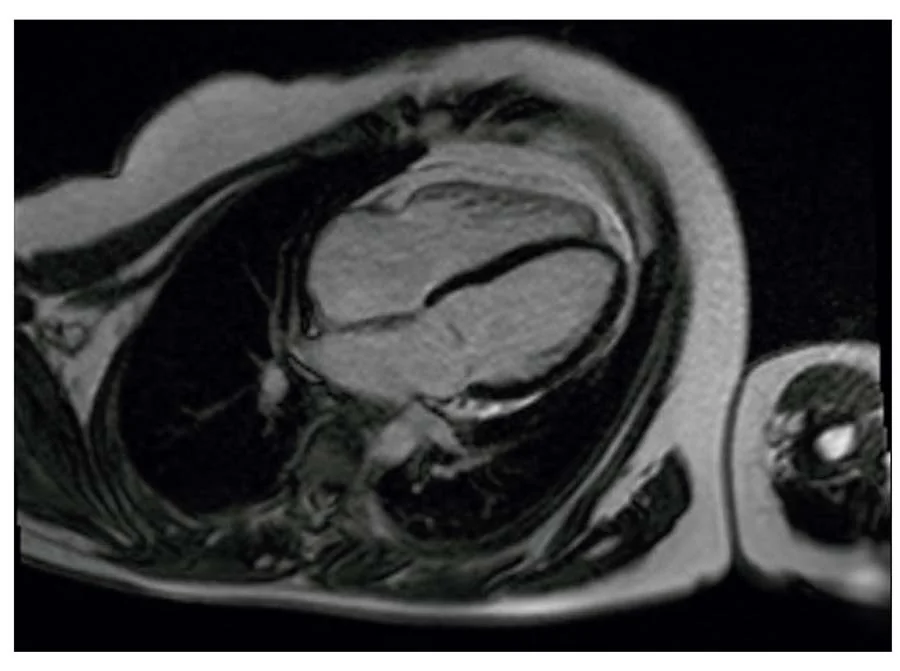
– Ressonância Magnética Cardíaca demonstrando pericárdio com discreto espessamento e derrame discreto a moderado.

Ao final da investigação, a paciente recebeu alta com tratamento empírico para possível pericardite viral com colchicina 0,5mg 2x ao dia e ibuprofeno 400mg 2x ao dia, com melhora parcial dos sintomas após 4 semanas.

Realizado novo ecocardiograma transtorácico de controle após 4 semanas e identificado aumento do derrame pericárdico (25mm), aumento da ecogenicidade e *swing heart*. (Figura 3). A partir desse achado, foi encaminhada à sala de emergência em bom estado geral, assintomática, com PA:111x59 mmHg, FC:58 bpm, FR:13 mrpm e saturação de oxigênio 97% em ar ambiente, exame físico normal e sem sinais clínicos de tamponamento cardíaco. O eletrocardiograma com único achado de supradesnivelamento do segmento ST, discreto e difuso, do tipo repolarização precoce - achado comum em jovens, inespecífico e que se manteve ao longo da internação (Figura 4).

**Figura 3:**
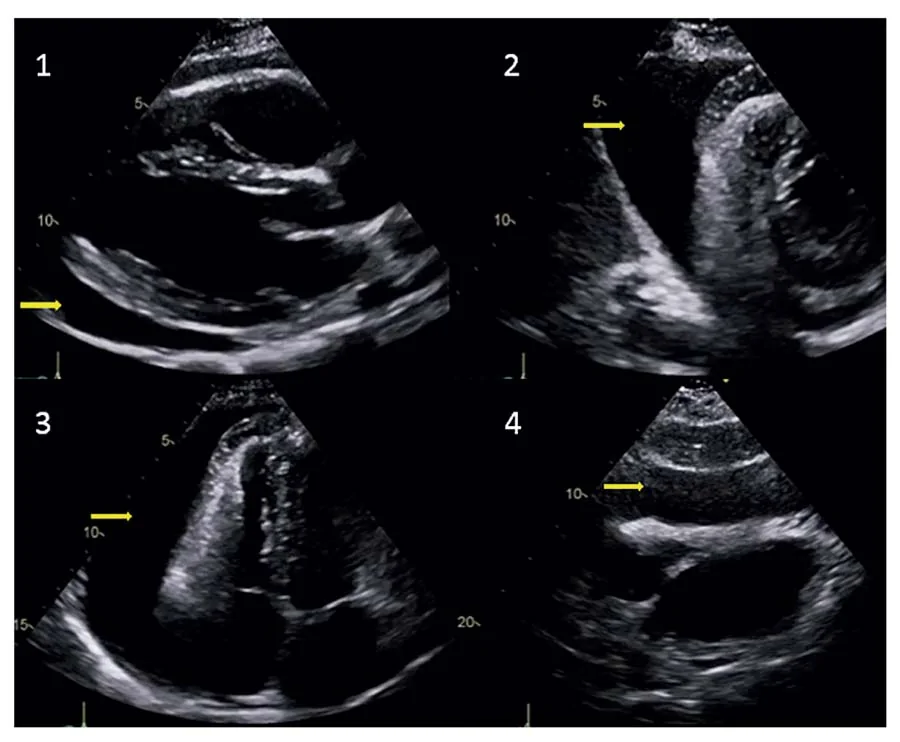
– Ecocardiograma transtorácico. 1- janela longitudinal eixo longo. 2- janela longitudinal eixo curto. 3- janela apical 4 câmaras. 4- janela subxifoide demonstrando restrição do enchimento do ventrículo direito. Setas: derrame pericárdico.

**Figura 4:**
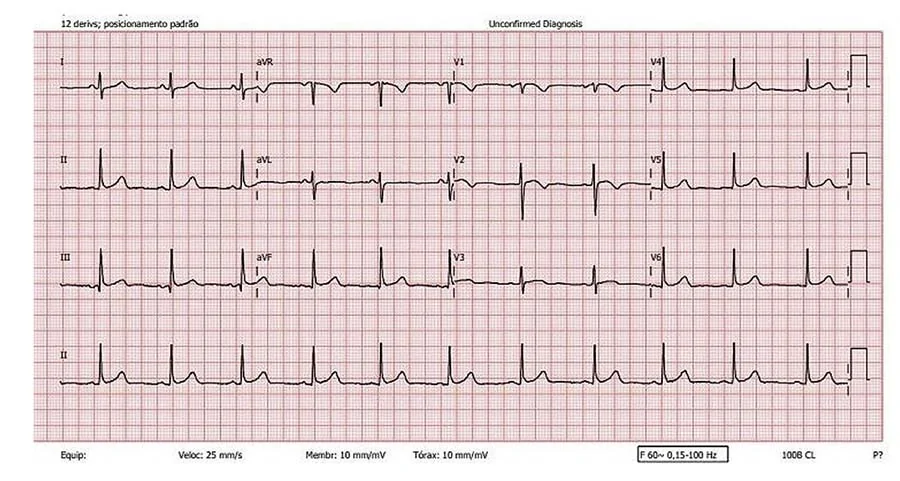
– Ritmo sinusal, eixo preservado, discreto supra de ST difuso e sem sinais sugestivos de tamponamento cardíaco.

Optado, portanto, pela realização de drenagem pericárdica de urgência com auxílio de vídeotoracoscopia, com saída de 400 mL de líquido amarelado, espesso e aspecto quiloso e visualizado pericárdio espessado e com aspecto fibrinoso. (Figura 5) A análise do líquido pericárdico revelou triglicerídeos de 984 mg/dl, colesterol total de 83 mg/dl, relação colesterol/triglicerídeos de 0,08 e predomínio linfocitário (89%), confirmando o diagnóstico de quilopericárdio. Perfil lipídico da paciente demonstrou CT:174 mg/Ll, TG:35 mg/dL, LDL-c:116 mg/dL e HDL-c:47 mg/dL. Demais análise do líquido pericárdio com pH:7,4, proteína:4,4 g/L, LDH:153 mg/dL, albumina: 2,9 g/L, amilase:93 mg/dL, glicose:90 mg/dL, pesquisa para tuberculose (ADA, BAAR e TR-TB) negativas, PCR-CMV, micológico direto, citologia oncótica e culturas todos negativos. A biópsia pericárdica demonstrou pericardite fibrinosa crônica em organização.

**Figura 5:**
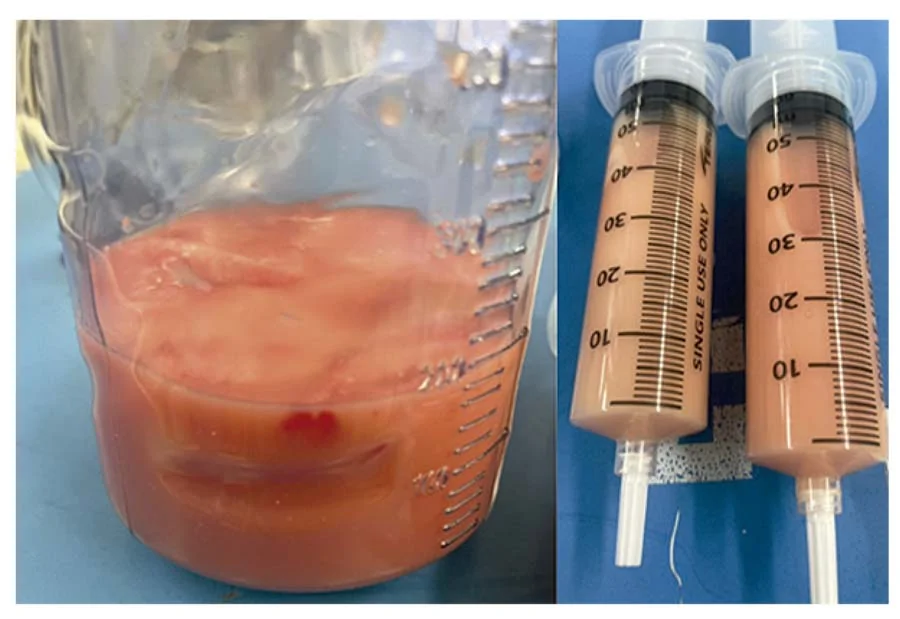
– Aspecto macroscópico do líquido pericárdico.

Após o estabelecimento do diagnóstico de quilopericárdio, a investigação etiológica incluiu tomografia computadorizada de tórax, sem alterações ou presença de massas, e linfocintilografia (Figura 6) – que demonstrou déficit de drenagem linfática funcional difuso, sem sinais de refluxo dérmico (sinal de linfedema), obstrução ou dilatação dos vasos linfáticos, drenagem anômala ou aumento de captação em regiões linfonodais.

**Figura 6:**
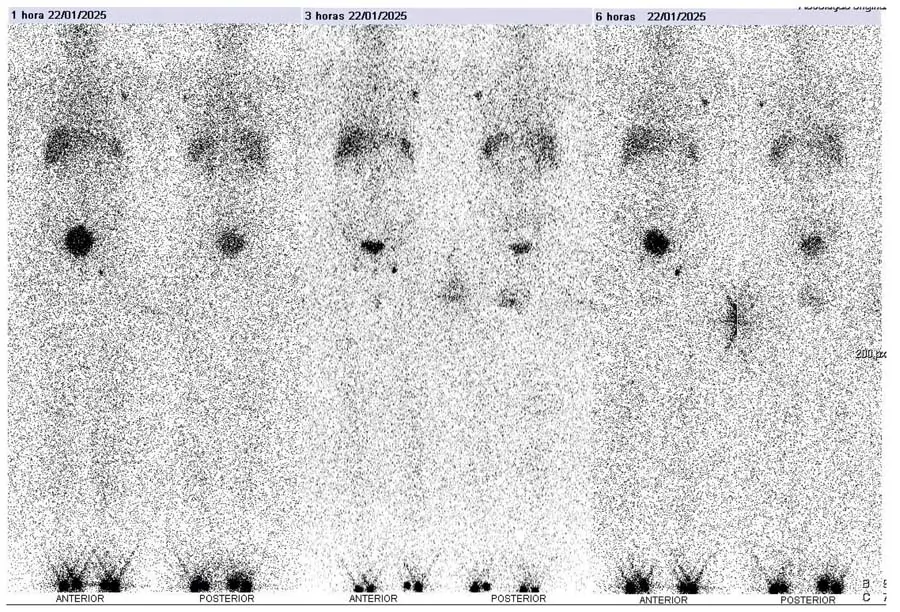
– Linfocintilografia é realizado com a injeção de radiofármaco de forma subcutânea entre os dedos das mãos e dos pés, sendo captado pelo sistema linfático e transportado ao longo dos vasos linfáticos; utilizado para avaliação de fluxo linfático, integridades dos vasos, linfonodos e refluxo dérmico (presente em casos de linfedema). A paciente em questão apresentou achados de déficit funcional da drenagem linfática de forma difusa. Sem evidências de obstruções, malformações congênitas ou fístulas do ducto linfático.

Excluídas causas secundárias, a paciente recebeu tratamento para quilopericárdio primário com dieta hipogordurosa e hiperproteica, associada à manutenção da drenagem pericárdica.

No pós- operatório, a PA oscilou entre 100-138x59-85 mmhg, a FC entre 51-95 bpm e a FR entre13-20 mrpm. Em avaliação diária, houve redução progressiva do débito do líquido pericárdico e queda sustentada dos níveis de triglicerídeos, sendo optado pela retirada do dreno após 10 dias do procedimento. O ecocardiograma transtorácico de controle não observou sinais de derrame pericárdico e paciente recebeu alta com 11 dias de internação hospitalar (Tabela 2).

**Tabela 2:** – Temporalidade dos eventos do caso

Evento	Tempo / Referência
Início dos sintomas	5 meses antes da 1º consulta
1º consulta ambulatorial	Dia 0 (referência)
ECG, RX de tórax, exames laboratoriais iniciais	Dia 2
Ecocardiograma transtorácico – derrame pericárdico moderado sem tamponamento	Dia 7
Internação hospitalar para investigação etiológica	Dia 13
Exames laboratoriais ampliados (reumatológicos, infecciosos, metabólicos)	Durante 1ª internação
Ressonância magnética cardíaca – função preservada, derrame pericárdico sem outras alterações	Durante 1ª internação
Alta hospitalar com tratamento empírico (colchicina+ ibuprofeno)	Dia 7 da 1ª internação
Retorno ambulatorial com ecocardiograma de controle – porá do derrame (25mm), swing heart	Semana 4 após alta
Encaminhamento á emergência	Semana 4
Drenagem pericárdica por videotoracoscopia – saída de 400 mL de líquido quiloso	Dia 1 da 2ª internação
Análise do líquido pericárdico – diagnóstico confirmado de quilopericárdio	Dia 2 da 2ª internação
TC de tórax e linfocintilografia – investigação etiológica complementar	Dia 3 da 2ª internação
Retirada do dreno pericárdico	Dia 10 do pós operatório
Ecocardiograma de controle – sem derrame	Dia 11 do pós operatório
Alta hospitalar	Dia 11 da 2ª internação
Seguimento ambulatorial	Após a alta, sem recorrência

ECG: eletrocardiograma; RX: raio x; TC: tomografia computadorizada.

Atualmente, a paciente segue em acompanhamento ambulatorial, assintomática e com evolução favorável e ecocardiogramas de controle após 3 e 6 meses e 1 ano sem novo episódio de derrame pericárdico.

## Discussão

O quilopericárdio é uma condição rara caracterizada pelo acúmulo de líquido linfático rico em triglicerídeos (quilo) no espaço pericárdico. Desde o primeiro relato de Groves^1^ em 1954, esse acometimento permanece pouco frequente, sendo a maior parte do conhecimento derivada de relatos de casos e pequenas séries, o que contribui para limitações na padronização diagnóstica e terapêutica. Uma metanálise realizada pela Mayo Clinic encontrou apenas 33 casos em uma década de estudo, entre o período de 1996 a 2006.^2^

A drenagem linfática do pericárdio é majoritariamente direcionada aos linfonodos mediastinais e peritraqueobrônquicos (75%) ou ascendendo às veias braquicefálicas (25%). Não há relato de vasos linfáticos que entrem diretamente no espaço pericárdio e que possam causar acúmulo de quilo na cavidade, todos permanecem no lado pleural do pericárdio fibroso. No entanto o mecanismo fisiopatológico mais provável para a presença de quilo no espaço pericárdio seria decorrente do refluxo dos vasos linfáticos mediastinais e traqueobrônquicos.^3^

O quadro clínico é variável e inespecífico. A maioria dos pacientes permanecem assintomáticos, enquanto outros apresentam dispneia progressiva, fadiga, intolerância ao esforço e, em casos graves, tamponamento cardíaco.^4,5^ De acordo com metanálises recentes aproximadamente 74% dos pacientes apresentavam sintomas no momento do diagnóstico, sendo a dispneia o mais comum e 38% com tamponamento cardíaco,^5^ com um intervalo entre o início dos sintomas até o diagnóstico de cerca de 13 anos.^2^ O curso clínico insidioso observado no presente caso está de acordo com esse padrão descrito na literatura.

A epidemiologia é incerta, com estudos mais atuais sugerindo até 60% dos casos são idiopáticos primários, acometendo predominantemente adultos jovens, sem clara predominância entre os sexos.^2,5^ As causas secundárias incluem cirurgia cardíaca ou torácica, trauma, neoplasias linfáticas ou outras neoplasias mediastinais (linfangioma mediastinal, disgerminoma mediastinal, carcinoma de células em anel de sinete), síndrome de Gorham, radioterapia, infecções, tuberculose, hipertensão pulmonar, trombose venosa e linfangiectasia mediastinal.^2,4^ Na ausência desses fatores, como no caso apresentado, o diagnóstico de quilopericárdio primário deve ser considerado como de exclusão. Acredita-se que o acúmulo de líquido linfático ocorra por comunicações anormais do ducto torácico com os vasos linfáticos pericárdicos ou pela elevação da pressão no ducto torácico, como o que ocorre na linfangiectasia.^2^

A investigação etiológica deve ser sistemática, especialmente nos casos sem causa aparente. As diretrizes da Sociedade Europeia de Cardiologia (ESC) recomendam investigação etiológica dirigida em derrames pericárdicos volumosos, recorrentes ou de etiologia indeterminada.^6^

O diagnóstico definitivo baseia-se na análise do líquido pericárdico. Níveis de triglicerídeos superiores a 500 mg/dl, relação colesterol/triglicerídeos inferior a 1, predomínio linfocitário e aspecto macroscópico leitoso ou amarelado são critérios amplamente aceitos para confirmação diagnóstica.^4,5,7^ Culturas geralmente são negativas, uma vez que o quilo possui propriedades bacteriostáticas.^4^ No nosso caso, os achados laboratoriais do líquido pericárdico foram compatíveis com a literatura, permitindo confirmação do diagnóstico.

Devido a alta incidência de tuberculose em nosso país, foram realizadas análises específicas e comparadas com achados da literatura. Derrame pericárdico por tuberculose geralmente apresenta-se como de aspecto hemorrágico, exsudativo, com alto teor de proteína, leucócitos com predomínio de linfócitos e monócitos.^8^ Nossos achados vão de encontro com a possibilidade de tuberculose, além dos resultados negativos para ADA, BAAR, TR-TB, cultura e análise histológica.

O ecocardiograma transtorácico é o exame inicial de escolha para avaliação do derrame pericárdico e repercussão hemodinâmica. Demais métodos de imagem complementares, como tomografia computadorizada e ressonância magnética cardíaca, são úteis para exclusão de neoplasias e outras causas secundárias.^4,6^ Outras formas de se avaliar a drenagem linfática é através da ingesta de corante Sudan III ou espécie de triglicerídeos marcado com iodo radioativo.^2^ A linfocintilografia e a linfangiografia auxiliam na identificação de alterações do ducto torácico ou comunicações linfáticas anômalas, sendo particularmente indicadas em casos recorrentes ou refratários.^2,7^

No presente relato a paciente apresenta déficit de drenagem linfática funcional, não demonstrando obstruções, comunicações anômalas ou refluxo dérmico. Esse achado foi interpretado como constitucional da paciente, visto que durante toda a internação não apresentou edema ou sinais de retenção hídrica.

O manejo terapêutico permanece desafiador devido à escassez de dados robustos. Estratégias conservadoras incluem drenagem pericárdica associada a dieta hipogordurosa, preferencialmente com triglicerídeos de cadeia média, suporte nutricional e, em casos selecionados, uso de octreotídeo.^4,7^Apesar disso, aproximadamente um terço dos pacientes evolui com falha do tratamento clínico, necessitando de intervenção cirúrgica, como ligadura do ducto torácico e criação de janela pericárdica.^4,5^ No caso apresentado, o tratamento com drenagem pericárdica associada a dieta hipogordurosa foi eficaz, com resolução clínica e laboratorial, sem recorrência no seguimento ambulatorial.

Outro achado de relevância é a presença de investigação da síndrome da Mayer–Rokitansky–Küster–Hauser a qual consiste por aplasia congênita do útero e da parte superior (⅔) da vagina em mulheres com desenvolvimento dos caracteres sexuais secundários normais. Esta síndrome é raramente associada a malformações cardíacas e quando presente envolvem desfechos letais ou graves, semelhante a síndrome de Holt-Oram ou velocardiofacial (CIA, CIV, bloqueio AV ou tetralogia de Fallot).^9^ Em nosso caso, mantivemos vigilância e orientação de seguir acompanhamento com especialidade ginecológica.

Em conjunto, este relato reforça a importância de considerar o quilopericárdio no diagnóstico diferencial de derrames pericárdicos e a necessidade de uma ampla variedade de exames para identificação da etiologia. O reconhecimento precoce, a análise detalhada do líquido pericárdico e a investigação etiológica sistemática são fundamentais para orientar o manejo e reduzir o risco de recorrência e complicações.^1,5,6^
