# Supplementary material for: Quilopericárdio Primário Idiopático
Source: Arq Bras Cardiol. 2026 Jul 31;123(7):e20260062. [Article in Portuguese] doi: 10.36660/abc.20260066 (PMC13668453; doi:10.36660/abc.20260066)
Supplement: Translation

# Translation: Idiopathic Primary Chylopericardium

**DOI:** 10.36660/abc.20260066i

**Authors:** Camila Helena Ruffo, Alfredo Aurélio Marinho Rosa, Guilherme Lima Favaro, Juliana Sayuri Maia Hirose, Raissa Matias Lewinter, Natan Melillo, Leonardo Izzo Silva, Ramez Anbar, Murilo Martins Coelho, Giuliano Generoso

**Affiliations:** 1 Brasil Hospital Sírio - Libanês, São Paulo, SP – Brasil

**Keywords:** Pericardium, Pericardial Effusion, Diagnostic Imaging

## Introduction

Chylopericardium is a rare entity, defined by the accumulation of chyle in the pericardial space, characterized by a high concentration of triglycerides and lymphocyte predominance. It was first described in 1954, and since then, this condition has remained infrequent, with most evidence coming from case reports and small series, which hinders early recognition and standardization of management.

The main causes include congenital lymphangiectasia, iatrogenic injury after cardiac or thoracic surgery, neoplasms, trauma, infection, radiation, pulmonary hypertension, subclavian vein thrombosis, and primary or idiopathic causes. Primary chylopericardium has a variable incidence in the literature and is a diagnosis of exclusion.

We report a case of primary chylopericardium in a young patient, emphasizing the diagnostic and therapeutic challenges of this rare condition.

## Description

A 31-year-old female patient, previously healthy, born and residing in São Paulo (SP), sought medical attention in an outpatient setting, complaining of progressive tiredness, fatigue, and exercise intolerance, which began 5 months prior. She reported dyspnea on minimal exertion, associated with dizziness, a feeling of imbalance, and daytime sweating, with gradual worsening over the months. She denied chest pain, fever, cough, weight loss, infectious symptoms, or a history of chest trauma or previous surgeries. Her personal history included bronchitis, and she was under investigation for primary amenorrhea, with suspected Mayer-Rokitansky-Küster-Hauser syndrome. She denied smoking, alcohol consumption, or illicit drug use.

On initial physical examination, the patient appeared to be in good general condition, with BP: 129x67 mmHg, HR: 65 bpm, RR: 16 brpm, oxygen saturation 97% on room air, afebrile, without signs of systemic congestion, with cardiovascular and respiratory examination showing no significant alterations.

The initial investigation included an electrocardiogram, laboratory tests (without specific findings), and a chest X-ray (Figure 1) showing a clear increase in the cardiac silhouette. Based on this finding, a transthoracic echocardiogram was requested, which revealed a moderate-volume pericardial effusion without signs of cardiac tamponade.

**Figure 1:** – Chest X-ray: increased cardiac silhouette.

Therefore, hospital admission was chosen for the investigation of the etiology of the pericardial effusion with laboratory tests such as rheumatological, infectious, and metabolic tests, as well as complementary imaging exams.

Further analysis showed normal blood count, electrolytes, urinalysis, renal, hepatic, and thyroid function, in addition to slightly altered inflammatory markers: CRP: 1.02 mg/dL (reference range <0.5 mg/dL) and ESR: 41 mm (reference range: 2-20 mm). Rheumatological evaluation demonstrated nonspecific and mild alterations, which do not determine a rheumatological diagnosis (Table 1).

**Table 1:** – Results of rheumatological investigation tests

Exams	Results	Reference value
ANA	negative	negative
RF	negative	Negative
C3	145 mg/dL	81-157 mg/dL
C4	46 mg/dL	13-39 mg/dL
ASO	592 IU/mL	<200UI/mL
Lupus anticoagulant	negative	negative
Anti-DsDNA	negative	negative
Anti-Jo 1	negative	negative
Anti-SCL 70	negative	negative
Anti-SSBLA	negative	negative
Anti-SSBR	negative	negative
Anti-Sm	negative	negative
Anti-RNP	negative	negative
Anticardiolipin	negative	negative
Alpha-1 acid glycoprotein	73 mg/dL	50-120 mg/dL
ANCA	negative	negative
Urinary creatinine	20 mg/kg/day	11-20 mg/kg/day
Protein electrophoresis		
Albumin	53%	55-66%
Beta-globulin	6.5%	3.2-6.5%
Gamma-globulin	21%	11-18%

ANA: antinucleus factor; RF: rheumatoid factor; C3: complement C3; C4: complement C4; ASO: antistreptolysin O; Anti-DSDNA: anti native DNA antibody; Anti-Jo1: anti-Jo1 antibody; Anti-SCL70: anti-SCL70 antibody; Anti-SSBLA: anti SSBLA antibody; Anti-SSARo: anti SSARo antibody; Anti-SM: anti SM antibody; Anti-RNP: anti RNP antibody; ANCA: anti-neutrophil cytoplasmic antibodies.

For better anatomical definition, she underwent cardiac magnetic resonance imaging, which demonstrated preserved biventricular systolic function, absence of myocardial fibrosis, and presence of pericardial effusion without other alterations (Figure 2).

**Figure 2:** – Cardiac Magnetic Resonance Imaging demonstrating pericardium with slight thickening and mild to moderate effusion.

At the end of the investigation, the patient was discharged with empirical treatment for possible viral pericarditis consisting of colchicine 0.5 mg twice daily and ibuprofen 400 mg twice daily, with partial improvement of symptoms after 4 weeks.

A follow-up transthoracic echocardiogram was performed after 4 weeks and identified increased pericardial effusion (25mm), increased echogenicity, and swing heart. (Figure 3). Based on this finding, she was referred to the emergency room in good general condition, asymptomatic, with BP: 111x59 mmHg, HR: 58 bpm, RR: 13 brpm, and oxygen saturation 97% on room air, normal physical examination, and no clinical signs of cardiac tamponade. The electrocardiogram showed a single finding of ST-segment elevation, mild and diffuse, of the early repolarization type - a common finding in young people, nonspecific, and which persisted throughout the hospitalization (Figure 4).

**Figure 3:** – Transthoracic echocardiogram. 1- Long-axis longitudinal window. 2- Short-axis longitudinal window. 3- Apical 4-chamber window. 4- Subxiphoid window demonstrating right ventricular filling restriction. Arrows: pericardial effusion.

**Figure 4:** – Sinus rhythm, preserved axis, slight diffuse ST elevation, and no signs suggestive of cardiac tamponade.

Therefore, an urgent pericardial drainage was performed with the aid of video-assisted thoracoscopy, resulting in the removal of 400 mL of thick, yellowish, chylous fluid, and a thickened, fibrinous pericardium was visualized. (Figure 5) Analysis of the pericardial fluid revealed triglycerides of 984 mg/dL, total cholesterol of 83 mg/dL, a cholesterol/triglyceride ratio of 0.08, and a predominance of lymphocytes (89%), confirming the diagnosis of chylopericardium. The patient’s lipid profile showed total cholesterol (TC): 174 mg/dL, triglycerides (TG): 35 mg/dL, LDL-c: 116 mg/dL, and HDL-c: 47 mg/dL. Further analysis of the pericardial fluid showed pH: 7.4, protein: 4.4 g/L, LDH: 153 mg/dL, albumin: 2.9 g/L, amylase: 93 mg/dL, and glucose: 90 mg/dL. Tuberculosis tests (ADA, AFB, and TB-TR) were negative. CMV-PCR, direct mycological examination, oncotic cytology, and cultures were all negative. Pericardial biopsy demonstrated chronic fibrinous pericarditis in the organizing phase.

**Figure 5:** – Macroscopic appearance of pericardial fluid.

Based on the confirmed diagnosis of chylopericardium, the etiological investigation included a chest computed tomography scan, which showed no abnormalities or masses, and lymphoscintigraphy (Figure 6), which demonstrated diffuse functional lymphatic drainage deficit, without signs of dermal reflux (a sign of lymphedema), obstruction or dilation of lymphatic vessels, anomalous drainage, or increased uptake in lymph node regions.

**Figure 6:** – Lymphoscintigraphy is performed by injecting a radiopharmaceutical subcutaneously between the fingers and toes, which is then absorbed by the lymphatic system and transported along the lymphatic vessels; it is used to assess lymphatic flow, vessel integrity, lymph nodes, and dermal reflux (present in cases of lymphedema). The patient in question presented findings of diffuse functional deficit of lymphatic drainage. There was no evidence of obstructions, congenital malformations, or lymphatic duct fistulas.

With secondary causes ruled out, the patient received treatment for primary chylopericardium with a low-fat, high-protein diet, along with maintenance of pericardial drainage.

In the postoperative period, blood pressure fluctuated between 100-138/59-85 mmHg, heart rate between 51-95 bpm, and respiratory rate between 13-20 breaths per minute. Daily assessments showed a progressive reduction in pericardial fluid output and a sustained decrease in triglyceride levels, leading to the decision to remove the drain 10 days after the procedure. The follow-up transthoracic echocardiogram showed no signs of pericardial effusion, and the patient was discharged after 11 days of hospitalization (Table 2).

**Table 2:** – Temporality of events in the case

Event	Time / Reference
Onset of symptoms	5 months before the first consultation
1st outpatient consultation	Day 0 (reference)
ECG, chest X-ray, initial laboratory tests	Day 2
Transthoracic echocardiogram – moderate pericardial effusion without tamponade	Day 7
Hospital admission for etiological investigation.	Day 13
Expanded laboratory tests (rheumatological, infectious, metabolic)	During the first hospitalization
Cardiac magnetic resonance imaging – preserved function, pericardial effusion without other abnormalities.	During the first hospitalization
Hospital discharge with empirical treatment (colchicine + ibuprofen)	Day 7 of the first hospitalization
Outpatient follow-up with echocardiogram – appearance of the effusion (25mm), swing heart.	Week 4 post-discharge
Referral to the emergency room	Week 4
Video-assisted pericardial drainage – drainage of 400 mL of chylous fluid.	Day 1 of the 2nd hospitalization
Analysis of pericardial fluid – confirmed diagnosis of chylopericardium	Day 2 of the 2nd hospitalization
Chest CT scan and lymphoscintigraphy – complementary etiological investigation	Day 3 of the 2nd hospitalization
Removal of the pericardial drain	Day 10 post-surgery
Follow-up echocardiogram – no stroke	Day 11 post-surgery
Hospital discharge	Day 11 of the 2nd hospitalization
Outpatient follow-up	After discharge, no recurrence.

ECG: electrocardiogram; CT: computed tomography.

Currently, the patient is under outpatient follow-up, asymptomatic, and with favorable progress. Follow-up echocardiograms were performed after 3, 6 months, and 1 year without a new episode of pericardial effusion.

## Discussion

Chylopericardium is a rare condition characterized by the accumulation of triglyceride-rich lymphatic fluid (chyle) in the pericardial space. Since Groves’ first report^1^ in 1954, this condition has remained infrequent, with most knowledge derived from case reports and small series, contributing to limitations in diagnostic and therapeutic standardization. A meta-analysis conducted by the Mayo Clinic found only 33 cases in a decade of study, between 1996 and 2006.^2^

Lymphatic drainage of the pericardium is primarily directed to the mediastinal and peritracheobronchial lymph nodes (75%) or ascending to the brachiocephalic veins (25%). There are no reports of lymphatic vessels entering directly into the pericardial space that could cause chyle accumulation in the cavity; all remain on the pleural side of the fibrous pericardium. However, the most likely pathophysiological mechanism for the presence of chyle in the pericardial space would be due to reflux from the mediastinal and tracheobronchial lymphatic vessels.^3^

The clinical picture is variable and nonspecific. Most patients remain asymptomatic, while others present with progressive dyspnea, fatigue, exercise intolerance, and, in severe cases, cardiac tamponade.^4,5^ According to recent meta-analyses, approximately 74% of patients presented with symptoms at the time of diagnosis, with dyspnea being the most common, and 38% with cardiac tamponade,^5^ with an interval between the onset of symptoms and diagnosis of approximately 13 years.^2^ The insidious clinical course observed in the present case is consistent with this pattern described in the literature.

The epidemiology is uncertain, with more recent studies suggesting that up to 60% of cases are primary idiopathic, predominantly affecting young adults, with no clear predominance between the sexes.2,5 Secondary causes include cardiac or thoracic surgery, trauma, lymphatic neoplasms or other mediastinal neoplasms (mediastinal lymphangioma, mediastinal dysgerminoma, signet ring cell carcinoma), Gorham’s syndrome, radiotherapy, infections, tuberculosis, pulmonary hypertension, venous thrombosis, and mediastinal lymphangiectasia.^2,4^ In the absence of these factors, as in the case presented, the diagnosis of primary chylopericardium should be considered one of exclusion. It is believed that the accumulation of lymphatic fluid occurs due to abnormal communications between the thoracic duct and the pericardial lymphatic vessels or due to increased pressure in the thoracic duct, as occurs in lymphangiectasia.^2^

Etiological investigation should be systematic, especially in cases with no apparent cause. The European Society of Cardiology (ESC) guidelines recommend targeted etiological investigation in large, recurrent, or undetermined pericardial effusions.^6^

The definitive diagnosis is based on analysis of the pericardial fluid. Triglyceride levels above 500 mg/dl, a cholesterol/triglyceride ratio below 1, lymphocyte predominance, and a milky or yellowish macroscopic appearance are widely accepted criteria for diagnostic confirmation.^4,5,7^ Cultures are usually negative, since chyle has bacteriostatic properties.^4^ In our case, the laboratory findings of the pericardial fluid were consistent with the literature, allowing confirmation of the diagnosis.

Due to the high incidence of tuberculosis in our country, specific analyses were performed and compared with findings from the literature. Pericardial effusion due to tuberculosis generally presents as hemorrhagic, exudative, with a high protein content, and leukocytes with a predominance of lymphocytes and monocytes.^8^ Our findings are consistent with the possibility of tuberculosis, in addition to negative results for ADA, AFB, TB-TR, culture, and histological analysis.

Transthoracic echocardiography is the initial examination of choice for evaluating pericardial effusion and hemodynamic repercussions. Other complementary imaging methods, such as computed tomography and cardiac magnetic resonance imaging, are useful for excluding neoplasms and other secondary causes.^4,6^ Other ways to assess lymphatic drainage include ingesting Sudan III dye or a type of triglyceride labeled with radioactive iodine.^2^ Lymphoscintigraphy and lymphangiography help identify alterations in the thoracic duct or anomalous lymphatic communications, being particularly indicated in recurrent or refractory cases.^2,7^

In this report, the patient presents with a functional lymphatic drainage deficit, showing no obstructions, abnormal communications, or dermal reflux. This finding was interpreted as constitutional to the patient, since she did not present with edema or signs of fluid retention throughout her hospitalization.

Therapeutic management remains challenging due to a scarcity of robust data. Conservative strategies include pericardial drainage combined with a low-fat diet, preferably with medium-chain triglycerides, nutritional support, and, in selected cases, the use of octreotide.^4,7^ Despite this, approximately one-third of patients experience failure of clinical treatment, requiring surgical intervention, such as thoracic duct ligation and creation of a pericardial window.^4,5^ In the case presented, treatment with pericardial drainage combined with a low-fat diet was effective, with clinical and laboratory resolution, without recurrence in outpatient follow-up.

Another relevant finding is the presence of investigation for Mayer–Rokitansky–Küster–Hauser syndrome, which consists of congenital aplasia of the uterus and the upper part (⅔) of the vagina in women with normal development of secondary sexual characteristics. This syndrome is rarely associated with cardiac malformations and, when present, involves lethal or severe outcomes, similar to Holt-Oram syndrome or velocardiofacial syndrome (ASD, VSD, AV block or tetralogy of Fallot).^9^ In our case, we maintained surveillance and advised the patient to continue follow-up with a gynecological specialist.

Taken together, this report reinforces the importance of considering chylopericardium in the differential diagnosis of pericardial effusions and the need for a wide variety of tests to identify the etiology. Early recognition, detailed analysis of pericardial fluid, and systematic etiological investigation are fundamental to guiding management and reducing the risk of recurrence and complications.^1,5,6^
